# CD30 + Primary intestinal T-cell lymphoma (unclassified) masquerading as chronic inflammation: a case report

**DOI:** 10.1186/s13000-022-01237-0

**Published:** 2022-06-25

**Authors:** Kashif Osmani, Eshana Shah, Bradley Drumheller, Shaun Webb, Manmeet Singh, Paul Rubinstein, John Patrick Galvin, Megan S. Lim, Carlos Murga-Zamalloa

**Affiliations:** 1 Department of Pathology, University of Illinois at Chicago, 840 S. Wood Street, 260 CMET, Chicago, USA; 2 Department of Internal Medicine, University of Illinois at Chicago, Chicago, USA; 3 Department of Pathology and Laboratory Medicine, University of Pennsylvania, Philadelphia, USA

**Keywords:** Primary intestinal, T-cell lymphoma, T-cell lymphoma NOS, Case report28

## Abstract

**Background:**

Primary intestinal T-cell lymphomas are uncommon malignancies that pose a diagnostic dilemma, because the clinical features and imaging findings commonly overlap with those encountered in inflammatory bowel diseases.

**Case presentation:**

The current clinical case report describes the clinical history, laboratory findings and histopathological analysis from a patient with non-specific gastrointestinal symptoms with a presumptive clinical diagnosis of inflammatory bowel disease, and two intestinal biopsy specimens with non-specific findings. Due to the persistent symptoms a third biopsy was consistent with primary intestinal T-cell lymphoma, a diagnosis that was elusive for months after the initial presentation. Clinical correlation with laboratory and histopathological findings is required to establish a definitive diagnosis and to further stratify the patients. In addition, the neoplastic cells featured partial expression of CD30, which had relevant therapeutic implications.

**Conclusions:**

Suspicion for an intestinal T-cell lymphoproliferative disorder should always exist in patients with persistent abdominal symptoms with no clear etiology. The current discussion provides a summary and review of the key diagnostic histological features for the classification of primary intestinal T-cell lymphomas. In addition, the discussion describes how specific the histological findings are relevant for the clinical management decisions.

## Introduction

Primary intestinal T-cell lymphomas account for less than 5% of the lymphomas of the gastrointestinal tract, and the clinical findings often overlap with those of inflammatory bowel disease. In addition, the histological findings often mimic those of non-specific chronic inflammation, or ulcer-site changes. Due to the rarity in incidence and the non-specific clinical and histological findings, the diagnosis is not straightforward. The current clinical case report describes the clinical presentation and histological findings from a patient with a diagnosis of primary intestinal T-cell lymphoma non-otherwise specified (ITCL-NOS), which remained occult for months despite two biopsies within the small intestine. In addition, the definitive diagnosis was only established after retrospective analysis from one of the biopsies that included areas of the submucosa, as the most superficial biopsies demonstrated only non-specific findings. Finally, expression of CD30 was identified in a percentage of the neoplastic cells, which has relevant therapeutic implications.

### Patient information

A 55-year-old male with a past medical history of chronic obstructive pulmonary disease and opioid use disorder presented to the emergency department with worsening abdominal distention. The patient reported unintentional weight loss of approximately 60 pounds over the last year, in addition to increasing difficulty with bowel movements. He denied melena, nausea, vomiting, fevers, flank pain, dysphagia, or odynophagia, and did not have any previous abdominal surgeries.

### Clinical findings

An initial computed tomography (CT) of the abdomen and pelvis with contrast demonstrated a small bowel obstruction with a transition point in the right-mid abdomen where the small bowel loop was noticeably thickened, raising concern for an inflammatory process. In addition, mesenteric adenopathy of uncertain etiology was also noted. The patient’s abdominal symptoms improved without any acute interventions and he was discharged.

Three weeks later, the patient arrived in the emergency department complaining of recurrent diffuse abdominal pain. A repeat CT of the abdomen revealed multiple dilated loops of bowel with several air-fluid levels. A subsequent colonoscopy revealed two polyps with no other abnormalities. A capsule study followed by a video capsule endoscopy showed erythematous gastritis, multiple small bowel ulcers, and an atrophic duodenum. Biopsy results from the polypoid lesions identified on colonoscopy showed non-specific chronic inflammation without any morphological evidence of inflammatory bowel disease (IBD). However, the patient underwent a double balloon push enterostomy two weeks post-discharge, which revealed multiple jejunal strictures and possible areas of inflammation suggestive of IBD. At this time, the patient was scheduled for outpatient therapeutic management of IBD.

Two months later, the patient arrived in the emergency department with excruciating abdominal pain with new onset nausea and vomiting. Abdominal x-rays showed signs concerning for pneumoperitoneum. During an emergent exploratory laparotomy, a perforated ulcer in the terminal ileum was identified and repaired and a Brooke loop ileostomy proximal to the repair was created. Biopsy results surrounding the perforation area demonstrated an ulcerated mucosa with non-specific chronic inflammation. The patient was discharged 21 days later following a lengthy stay in the surgical intensive care unit.

### Diagnostic assessment

**Fig. 1:**
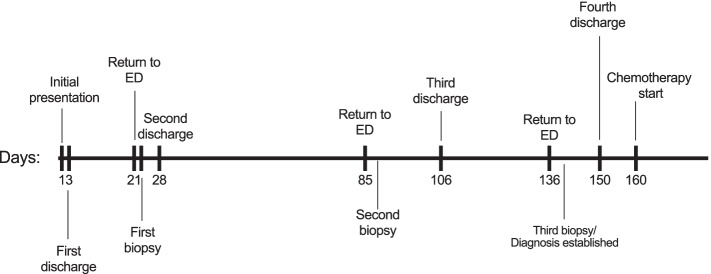
Timeline of relevant clinical events

**Fig. 2:**
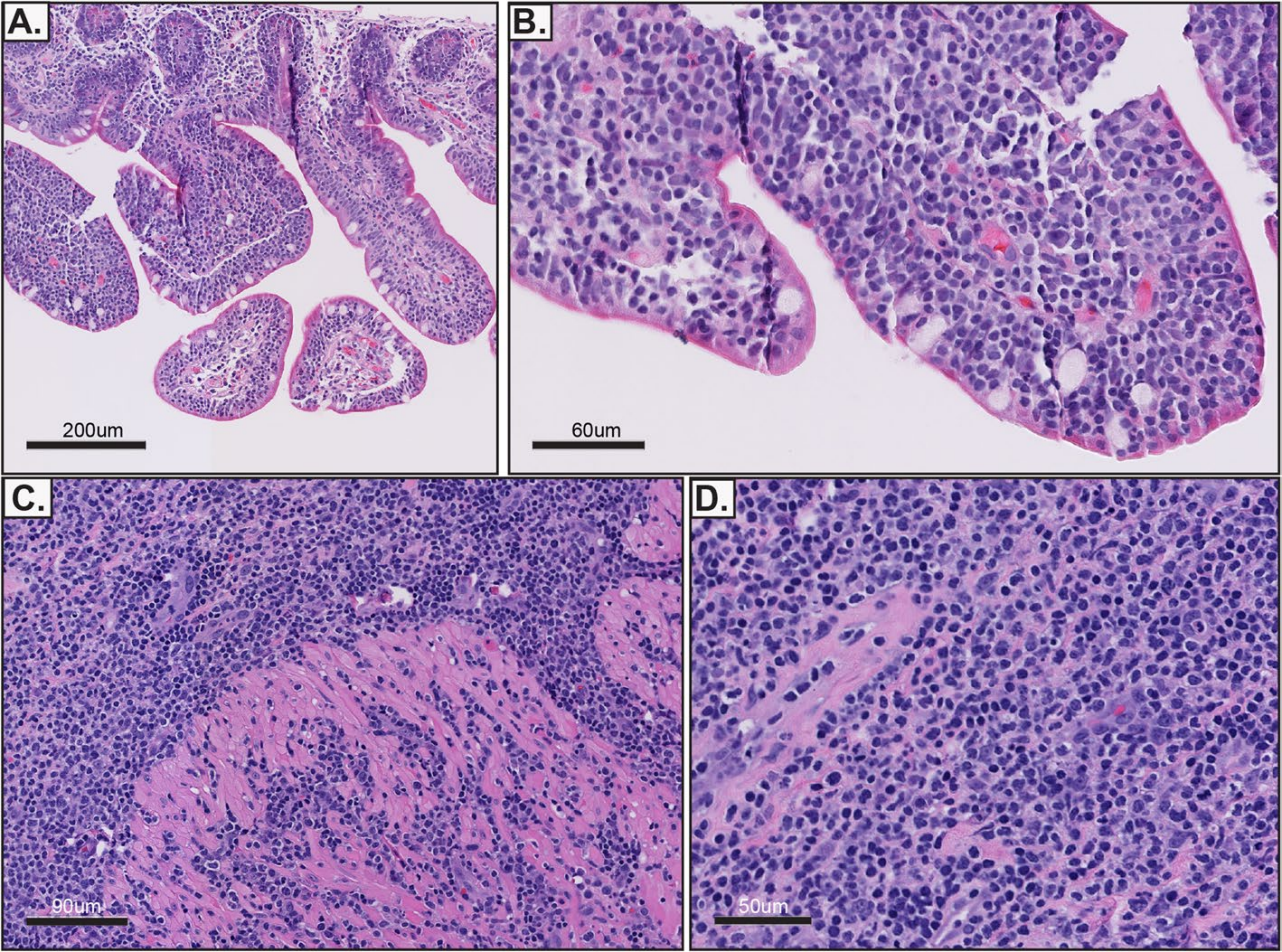
Representative images from biopsies of the small intestine. **A**, **B** Intact intestinal villi can be appreciated with extensive epitheliotropism of small lymphocytes. **C**, **D** The cytological atypia is more evident within the intestinal submucosa (**D**). Hematoxylin and eosin stain (**A**-**D**)

**Fig. 3:**
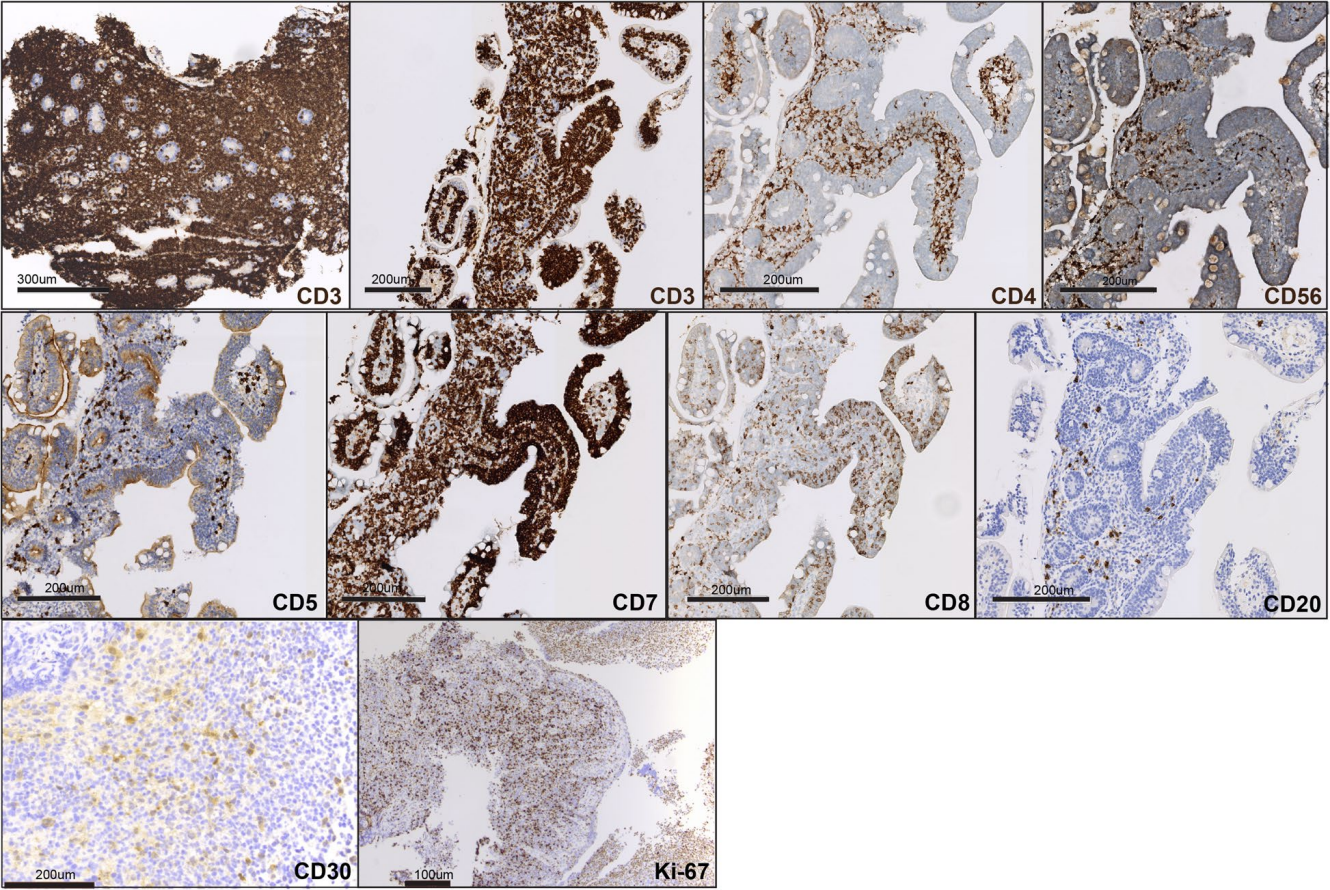
Immunohistochemical characterization of the atypical lymphocytic infiltrates. Each of the immunostains analyzed is indicated in the corresponding image

A month later, the patient was re-hospitalized for non-specific abdominal symptoms, with increased ostomy output. An ileoscopy performed at the end of this hospital stay showed the entire ileum exhibiting scalloped mucosa with blunted villi, and a biopsy was performed. A summary timeline of the relevant clinical events is included (Fig. 1). The latest biopsy evaluation from the terminal ileum mucosa demonstrated extensive lymphocytic infiltrates with epitheliotropism with no definitive atypical morphological features, which were suspicious for a lymphoid neoplasm, and the hematopathology service was consulted (Fig. 2). The two initial biopsies were evaluated by general surgical pathologists, and did not include immunohistochemical stains to characterize the lymphocytic infiltrates. Therefore, those prior intestinal biopsies were re-evaluated by an hematopathologist. The original biopsies revealed dense atypical lymphoid infiltrates present within the lamina propria. A subset of these atypical lymphocytes were medium to large in size, with irregular nuclear contours (Fig. 2). Immunohistochemical stains demonstrated that the lymphoid infiltrates were positive for CD3, CD4, CD8, CD103, TCR betaF1 (focally), CD30 (approximately 20% of cellularity), and were negative for CD20, CD79a, TCR g/d, CD5 and CD56 (Fig. 3). EBER in-situ hybridization for EBV was negative. The Ki-67 proliferation index was approximately 30%. T-cell receptor rearrangement studies were positive for a clonal T-cell population. A final diagnosis of intestinal T-cell lymphoma not otherwise specified (ITCL-NOS) was rendered.

### Therapeutic intervention

Patient was started with the first cycle of CHOP, pending insurance approval for BV-CHP.

### Follow-up and outcomes

To the date of publication submission, the patient received one cycle of CHOP with marked improvement in ostomy output with no blood in the outputs, and no other critical events before subsequent follow-up office visit. No significant adverse outcomes were reported by the patient after chemotherapy was started.

## Discussion

Although the initial clinical presentation and imaging findings were more consistent with an inflammatory bowel disease, the initial histological findings did not support such type of diagnosis. The typical morphologic features of small intestinal Crohn’s disease that can be seen in biopsy specimens include lamina propria involvement by increased acute and chronic inflammation, associated with neutrophilic cryptitis and crypt abscess, along with surface erosion and/or ulceration. In addition, features of chronic mucosal injury typically seen in this setting include villous and/or glandular atrophy, cystic change, crypt branching, pseudopyloric metaplasia, fibrosis, and granulomas, which often occur in a patchy distribution. These characteristic features of small intestinal Crohn’s disease, in particular the chronic mucosal injury related changes as noted above, were not well represented within the small intestinal biopsy specimens reviewed, making a sole diagnosis of small intestinal Crohn’s disease less likely. On the other hand, the dense atypical lymphoid infiltrates seen within the lamina propria associated with epitheliotropism, in the absence of significant chronic mucosal architectural changes, raises concern for a lymphoproliferative neoplasm and could help distinguish a lymphoproliferative neoplasm from small intestinal Crohn’s disease, in particular when significant epitheliotropism is identified [1]. It is recognized however, that there can be overlap in the clinical presentation, endoscopic findings and morphologic appearance seen in intestinal chronic inflammatory disorders and intestinal lymphoproliferative neoplasms leading to diagnostic challenges, although, the presence of a dense atypical lymphoid infiltrate with significant epitheliotropism should raise the possibility of a lymphoproliferative neoplasm in the differential diagnostic workup [1]. Lastly, the patchy disease distribution, the possibility of an IBD-associated lymphoproliferative neoplasm and the typical initial endoscopic mucosal biopsy specimen type, which can be somewhat superficial in nature (e.g. versus an intestinal resection specimen allowing for transmural microscopic evaluation), can further add to the diagnostic challenges in this differential diagnostic setting.

Subsequent biopsies revealed extensive superficial infiltrates with a mild degree of cytological atypia. In that setting, the specific classification of the atypical lymphoid infiltrates would be more difficult. Importantly, the biopsy that was performed at the site of the terminal ileum perforation included an area that was deep into the submucosa, and the degree of cytological atypia was more evident (Fig. 2). This degree of cytologic atypia and the elevated proliferation index supported an aggressive T-cell lymphoma and excluded the possibility of indolent intestinal T-cell lymphoma [1, 2]. Indolent intestinal T-cell lymphomas typically do not exhibit a Ki-67 proliferation index that is more than 5% [2]. The presence of epitheliotropism with absence of CD56 also do not support a diagnosis of NK-cell enteropathy [3]. Furthermore, the presence of a clonal rearrangement in the T-cell receptor is not usually identified in NK-cell enteropathy [3]. Evaluation for the presence of EBV is important. If positive in immunocompetent patients, the possibility of extranodal NK/T-cell lymphoma, nasal type can be considered. However in this case EBER in-situ hybridization for EBER was negative. There were no definitive clinical, serological or histological findings that supported a diagnosis of celiac disease, therefore a diagnosis of enteropathy-associated T-cell lymphoma (EATL) was not considered [4]. Notably, the tumor was negative for CD56, CD8-positive tumor cells were rare, and there was focal TCRβF1 expression. This absent cytotoxic immunophenotype that is characteristic of MEITL precludes this specific diagnosis, even in the presence of the monomorphic appearance and epitheliotropism [5, 6]. The overall discussed histologic and immunophenotypic features here lead to the diagnosis of intestinal T cell lymphoma, not otherwise specified (Table 1).

**Table 1:** Differential diagnosis to Intestinal T cell lymphomas

	**NK-cell enteropathy**	**Indolent T cell LPD of the GI tract**	**EATL**	**MEITL**
Epidemiology	*Unknown*	*Unknown*	Northern Europe	Asian, Hispanic
Associations	*Unknown*	*Unknown*	Celiac, HLA-DQ2, HLA-DQ8	*Unknown*
Location	Stomach, small intestine, colon	Small intestine, colon, others	Small intestine	Small intestine
Histology	Medium to large in size, with mild cytological atypia. Destruction of adjacent glands may be present at advanced stages Epitheliotropism is usually absent.	Small and monotonous lymphocytes, with none to mild cytological atypia. Non-destructive. Occasional epitheliotropism.	Pleomorphic. Medium to large with cytological atypia. Epitheliotropism is usually present. Angiodestruction may be present.	Monomorphic medium cells. Epitheliotropism usually present.
Phenotype	cCD3 + ^a^ CD8- CD5- CD7 + CD4- CD56 + TIA1 + EBER(ish)- Granzyme B + Low Ki-67(< 25%)	CD2 + CD3 + CD5 ± CD7 ± CD8 + > CD4 + ^c^ CD56- TIA1-/ + Granzyme B- EBER(ish) - Low Ki-67 (< 10%)	CD3 + CD4- CD5- CD7 + CD8-/ + ^d^ CD56- CD103 + CD30 ± TIA-1 + , Granzyme B + High Ki-67 (> 50%)	CD3 + CD5- CD8 + > CD4 + CD56 + ^b^ CD30- CD103 + TIA1 + Granzyme B + EBER(ish)- High Ki-67 (> 50%). MATK + ^#^
TCR expression	Negative	Alpha beta (αβ)	Alpha beta (αβ) > gamma delta (ɣδ)	Gamma delta (ɣδ) > Alpha beta (αβ)
Molecular	TCR polyclonal	STAT3-JAK2 fusion	STAT5B, JAK3, GNAI2 Gains of 1q and 5q	SETD2, STAT5B, JAK3, GNAI2 Gains of MYC

^a^ Concurrent flow cytometry analysis demonstrates that CD3 expression is cytoplasmic

^b^ Majority of tumors (~ 80%) are CD8 + , a small subset of cases (9–18%) can be negative for CD56

^c^ Majority of the cases are CD8 + (~ 80%), a minority of the cases are either CD4 + or double CD4/CD8 negative in similar proportions

^d^ Approximately 30% of the cases are CD8 + , majority of the cases are CD4-/CD8-. ^#^MATK/Lsk nuclear expression is detected in majority of MEITL cases and in NK/T cell lymphomas, whereas EATL cases only feature cytoplasmic staining

It is critical to discriminate and correctly diagnose an indolent disease from its more aggressive kindred, as it has significant implications on therapy and prognosis. Intestinal T-cell lymphomas are, in general, aggressive malignancies with a median overall survival (OS) of less than one year and a 5-year overall survival of 20%. Due to the aggressiveness of both MEITL and EATL, there have been efforts to improve treatment options [6]. However, due to the rarity of intestinal T-cell lymphomas, randomized controlled studies are not feasible [7]. Prospective studies have attempted to compare the efficacy of CHOP (cyclophosphamide, doxorubicin, vincristine, prednisone), CHOEP (cyclophosphamide, doxorubicin, vincristine, etoposide, prednisone) and IVE/MTX (ifosfamide, vincristine, etoposide/methotrexate). Results of these studies did not prove any regimen to be superior in regard to prolonging overall survival [4]. Analyses indicate that low-risk IPI, early stage and consolidative high-dose chemotherapy with autologous stem cell rescue improve outcomes [8]. ITCL-NOS was recently categorized and is without a preceding counterpart (such as MEITL and type II EATL), therefore, outcomes data are emerging. However, we know that these cases mimic the systemic counterpart and have an aggressive clinical course with dismal prognosis. In contrast, Indolent T-cell lymphoproliferative disorder of the gastrointestinal tract (ITLPD-GIT) has a good prognosis and can often be managed without chemotherapy [1, 2].

Approximately 20% of the tumor cells featured expression of CD30. The anti-CD30 drug conjugate Brentuximab vedotin (BV) was approved in 2018 for previously untreated CD30 + peripheral T-cell lymphomas after ECHELON-2, a multi-center randomized phase 3 study of BV-CHP, established the benefit of adding targeted CD30 + therapy. Unfortunately, only 2 patients with intestinal T-cell lymphoma were enrolled in ECHELON-2 but case reports exist on the use of BV in intestinal T-cell lymphomas [9]. There are numerous ongoing trials to further evaluate the efficacy of BV as monotherapy or combination therapy in PTCLs, including in low CD30-expressing PTCL (NCT02588651, NCT04569032). Although we cannot comment on the use of BV in intestinal T-cell lymphoma per se, we can extrapolate data on the role of CD30 expression in responses from knowledge in other PTCL subtypes.

Early studies in R/R PTCL used a generous CD30 cutoff of 1%, and the ECHELON-2 used a 10% threshold. Of 9 patients in ECHELON-2 who ultimately had CD30 < 10% upon central review, 7 responded including 5 with CR. In additional studies in PTCL and MF, one-third to two-third of patients responded when CD30 + expression was < 10%, including some who had no CD30 expression [10]. On the other hand, in one study of patients with high CD30 expression, defined as > 30%, response rates were only 48% (which is lower than in ECHELON-2) and no association was found between CD30 value and response or survival. These results underscore that anti-CD30 therapy likely has pleiotropic side effects including possibly on the tumor microenvironment to effect responses.

## Conclusions

Intestinal lymphomas account for approximately 5 to 10% of neoplasms from the gastrointestinal tract, and less than 10% of those are classified as T-cell lymphomas. The clinical presentation and imaging findings of primary intestinal T-cell lymphomas mimic those of inflammatory bowel diseases, and the diagnosis can be difficult to establish. Consultation with hematopathologists and characterization of the lymphoid component is warranted in cases where dense lymphoid infiltrates are present, and are not consistent with those of inflammatory bowel disease. The classification of intestinal T-cell lymphomas requires a complete clinical, and serological evaluation for the presence or absence of gluten enteropathy, in addition to histological evaluation that assesses for cytological atypia, proliferation index, expression of cytotoxic markers, and classification of the cell-of-origin of the tumor including (alpha beta/gamma delta subtype). Intestinal T-cell lymphoma NOS is not an specific entity, it rather represents all the tumors that fail current classification schemes, and very few cases are reported within the literature, and the clinical management of intestinal T-cell lymphoma NOS is similar as systemic aggressive counterparts.

## Data Availability

Not applicable.

## Abbreviations

CT: Computed Tomography
IBD: Inflammatory bowel disease
ITCL-NOS: Intestinal T-cell lymphoma not otherwise specified
TCR: T-cell receptor
NK: Natural Killer
EATL: Enteropathy-associated T-cell lymphoma
MEITL: Monomorphic epitheliotropic intestinal T-cell lymphoma
OS: Overall Survival
MF: Mycosis fungoides
IPI: International Prognostic Index
CHOP: Cyclophosphamide, doxorubicin, vincristine, prednisone
CHOEP: Cyclophosphamide, doxorubicin, vincristine, etoposide, prednisone
IVE/MTX: Ifosfamide, vincristine, etoposide/methotrexate
ITLPD-GIT: Indolent T-cell lymphoproliferative disorder of the gastrointestinal tract
BV: Brentuximab vedotin
BV-CHP: Brentuximab vedotin, Cyclophosphamide, Doxorubicin, Prednisone
PTCL: Peripheral T-cell lymphoma
CR: Complete response
R/R: Refractory/resistant
